# CVIT expert consensus document on primary percutaneous coronary intervention (PCI) for acute myocardial infarction (AMI) in 2018

**DOI:** 10.1007/s12928-018-0516-y

**Published:** 2018-03-29

**Authors:** Yukio Ozaki, Yuki Katagiri, Yoshinobu Onuma, Tetsuya Amano, Takashi Muramatsu, Ken Kozuma, Satoru Otsuji, Takafumi Ueno, Nobuo Shiode, Kazuya Kawai, Nobuhiro Tanaka, Kinzo Ueda, Takashi Akasaka, Keiichi Igarashi Hanaoka, Shiro Uemura, Hirotaka Oda, Yoshiaki Katahira, Kazushige Kadota, Eisho Kyo, Katsuhiko Sato, Tadaya Sato, Junya Shite, Koichi Nakao, Masami Nishino, Yutaka Hikichi, Junko Honye, Tetsuo Matsubara, Sumio Mizuno, Toshiya Muramatsu, Taku Inohara, Shun Kohsaka, Ichiro Michishita, Hiroyoshi Yokoi, Patrick W. Serruys, Yuji Ikari, Masato Nakamura, The Task Force on Primary Percutaneous Coronary Intervention (PCI) of the Japanese Cardiovascular Interventional Therapeutics (CVIT)

**Affiliations:** 1 Department of Cardiology, Fujita Health University School of Medicine, Toyoake, Japan; 2 Academic Medical Center, University of Amsterdam, Amsterdam, The Netherlands; 3 Thoraxcenter, Erasmus MC, Erasmus University, Rotterdam, The Netherlands; 4 Department of Cardiology, Aichi Medical University, Nagakute, Japan; 5 Department of Cardiology, Teikyo University Hospital, Tokyo, Japan; 6 Higashi Takarazuka Satoh Hospital, Takarazuka, Japan; 7 Division of Cardio-vascular Medicine, Department of Internal Medicine, Kurume University School of Medicine, Kurume, Japan; 8 Division of Cardiology, Hiroshima City Hiroshima Citizens Hospital, Hiroshima, Japan; 9 Department of Cardiology, Chikamori Hospital, Kochi, Japan; 10 Division of Cardiology, Tokyo Medical University Hachioji Medical Center, Tokyo, Japan; 11 Rakuwakai Kyoto Cardiovascular Intervention Center, Rakuwakai Marutamachi Hospital, Kyoto, Japan; 12 Department of Cardiovascular Medicine, Wakayama Medical University, Wakayama, Japan; 13 Hanaoka Seisyu Memorial Cardiovascular Clinic, Sapporo, Japan; 14 Cardiovascular Medicine, Kawasaki Medical School, Kurashiki, Japan; 15 Department of Cardiology, Niigata City General Hospital, Niigata, Japan; 16 Department of Cardiology, Katta General Hospital, Shiroishi, Japan; 17 Kurashiki Central Hospital, Kurashiki, Japan; 18 Kusatsu Heart Center, Kusatsu, Japan; 19 Sapporo Cardiovascular Clinic, Sapporo, Japan; 20 Saka General Hospital, Shiogama, Japan; 21 Cardiology Division, Osaka Saiseikai Nakatsu Hospital, Osaka, Japan; 22 Division of Cardiology, Saiseikai Kumamoto Hospital Cardiovascular Center, Kumamoto, Japan; 23 Division of Cardiology, Osaka Rosai Hospital, Sakai, Japan; 24 Department of Cardiovascular Medicine, Saga University, Saga, Japan; 25 Kikuna Memorial Hospital, Yokohama, Japan; 26 Toyohashi Heart Center, Toyohashi, Japan; 27 Murakami Clinic, Murakami, Japan; 28 Tokyo General Hospital, Tokyo, Japan; 29 Keio University School of Medicine, Tokyo, Japan; 30 Yokohama Sakae Kyosai Hospital, Federation of National Public Service Personnel Mutual Associations, Yokohama, Japan; 31 Cardiovascular Center, Fukuoka Sanno Hospital, Fukuoka, Japan; 32 Imperial College, London, UK; 33 Department of Cardiology, Tokai University School of Medicine, Kanagawa, Japan; 34 Division of Cardiovascular Medicine, Ohashi Medical Center, Toho University School of Medicine, Tokyo, Japan

**Keywords:** ST elevation acute myocardial infarction, Acute coronary syndrome, Plaque rupture, Plaque erosion, Percutaneous ventricular assist devices, Guideline

## Abstract

While primary percutaneous coronary intervention (PCI) has significantly contributed to improve the mortality in patients with ST segment elevation myocardial infarction even in cardiogenic shock, primary PCI is a standard of care in most of Japanese institutions. Whereas there are high numbers of available facilities providing primary PCI in Japan, there are no clear guidelines focusing on procedural aspect of the standardized care. Whilst updated guidelines for the management of acute myocardial infarction were recently published by European Society of Cardiology, the following major changes are indicated; (1) radial access and drug-eluting stent over bare metal stent were recommended as Class I indication, and (2) complete revascularization before hospital discharge (either immediate or staged) is now considered as Class IIa recommendation. Although the primary PCI is consistently recommended in recent and previous guidelines, the device lag from Europe, the frequent usage of coronary imaging modalities in Japan, and the difference in available medical therapy or mechanical support may prevent direct application of European guidelines to Japanese population. The Task Force on Primary Percutaneous Coronary Intervention of the Japanese Association of Cardiovascular Intervention and Therapeutics (CVIT) has now proposed the expert consensus document for the management of acute myocardial infarction focusing on procedural aspect of primary PCI.

## Introduction

In ST segment elevation myocardial infarction (STEMI), primary PCI has been shown to reduce cardiac events, to convey earlier discharge and to contribute to hemodynamic stabilization in cardiogenic shock and subsequently to become a standard care in Japan [1–19]. Despite a high number of available facilities providing primary PCI in Japan, there are no guidelines focusing on procedural aspect of standardized care, which may further improve the quality of our practice.

Recently, updated guidelines for the management of acute myocardial infarction (AMI) were published by European Society of Cardiology (ESC) [20]. As major changes, (1) radial access and drug-eluting stent (DES) over bare metal stent (BMS) were recommended as Class I indication, (2) complete revascularization before hospital discharge (either immediate or staged) is now considered as Class IIa recommendation. The primary PCI is consistently recommended in the updated guidelines as well as the previous guidelines [21].

However, the device lag between Europe and Japan, the difference in available medical therapy and mechanical support may prevent direct application of European guidelines to Japanese population (Tables 1, 2). Therefore, the Task Force on Primary Percutaneous Coronary Intervention (PCI) of the Japanese Association of Cardiovascular Intervention and Therapeutics (CVIT) summarized the expert consensus for the management of acute myocardial infarction, mainly focusing on procedural aspect.

**Table 1:** Major differences in available medication and mechanical devices

	Europe	Japan
Glycoprotein IIb/IIIa inhibitors	Tirofiban, eptifibatide, and abciximab are available	GP IIb/IIIa inhibitors are not available
P2Y12 inhibitors	The preferred P2Y12 inhibitors are prasugrel [60 mg loading dose and 10 mg maintenance dose once daily per os (p.o.)] or ticagrelor (180 mg p.o. loading dose and 90 mg maintenance dose twice daily)	Both prasugrel and ticagrelor are available, but the dose in prasugrel is different. [20 mg loading dose and 3.75 mg maintenance dose once daily per os]
Mechanical LV assist devices	Intra-cardiac axial flow pump (i.e. Impella) and intra-aortic balloon pump are available	Intra-aortic balloon pump is used. Intra-cardiac axial flow pump (i.e. Impella) is not widely available but used in limited institutions

**Table 2:** Major CE approved DES and their availability in Japan

DES	Stent platform	Polymer coating	Drug	Availability in Japan
Based on durable polymer coatings				
DESyne Nx	Cobalt-chrome	PBMA	Novolimus	No
Promus element	Platinum-chrome	PBMA and PVDF-HFP	Everolimus	Yes
Resolute	Cobalt-chrome	PBMA, PHMA, PVP, and PVA	Zotarolimus	Yes
STENTYS	Nitinol	PSU and PVP	Paclitaxel	No
Xience	Cobalt-chrome	PBMA and PVDF-HFP	Everolimus	Yes
Based on biodegradable polymer coatings				
Axxess	Nitinol	PDLLA	Biolimus A9	No
Biomatrix	Stainless steel	PDLLA	Biolimus A9	No
BioMime	Cobalt-chrome	PLLA and PLGA	Sirolimus	No
Combo	Stainless steel	PDLLA and PLGA + additional coating with anti-CD34	Sirolimus	No
DESyne BD	Cobalt-chrome	PLLA	Novolimus	No
Infinnium	Stainless steel	PLLA, PLGA, PCL, and PVP	Paclitaxel	No
MiStent	Cobalt-chrome	PLGA	Crystalline sirolimus	No
Nobori	Stainless steel	PDLLA	Biolimus A9	Yes
Orsiro	Cobalt-chrome	PLLA	Sirolimus	Yes
Supralimus Core	Cobalt-chrome	PLLA, PLGA, PCL, and PVP	Sirolimus	No
Synergy	Platinum-chrome	PLGA	Everolimus	Yes
Ultimaster	Cobalt-chrome	PDLLA and PCL	Sirolimus	Yes
Yukon Choice PC	Stainless steel	PDLLA	Sirolimus	No
Polymer-free				
Amazonia Pax	Cobalt-chrome	–	Paclitaxel	No
BioFreedom	Stainless steel	–	Biolimus A9	Yes
Cre8	Cobalt-chrome	–	Sirolimus	No
Yukon Choice PF	Stainless steel	–	Sirolimus	No
Coroflex ISAR	Cobalt-chrome	–	Sirolimus	No

### Specific differences between Japan and Europe (Table 1)

For example, glycoprotein (GP) IIb/IIIa inhibitors are not available in Japan, where thrombus aspiration is still a first choice of strategy of treatment of AMI.

Currently preferred oral P2Y12 inhibitors in acute coronary syndrome (ACS) in Europe are prasugrel and ticagrelor. Although ticagrelor became available recently in Japan, it was approved in 2016 and put on the market in February 2017, with a 7-year lag from the approval in Europe. In addition, dose difference in P2Y12 inhibitors between Japan and Europe may cause different anti-thrombotic benefit/bleeding risk profile. Intravenous cangrelor is not approved in Japan, while its use may be considered in patients not pre-treated with oral P2Y12 inhibitors at the time of PCI or in those who are considered unable to absorb oral agents in Europe.

LV assist devices and ECMO are increasingly popular managing patients with cardiogenic shock in Europe although they have not been sufficiently evaluated in clinical trials, while the use of IABP has not met prior expectations of benefit [22, 23]. In contrast, left ventricular assist devices (LVADs, i.e. intra-cardiac axial flow pumps and arterial-venous extracorporeal membrane oxygenation) are not widely available in Japanese institutions, while percutaneous ventricular assist devices (Impella) has recently been approved in limited institution in Japan where we largely rely on intra-aortic balloon pump as a mechanical support.

Regarding intravascular imaging devices, intravascular ultrasound and optical coherence tomography during PCI are routinely reimbursed in Japan. On the contrary to the situation in Europe and United States of America, its use is not restricted in selected cases such as unprotected left main lesions or stent failure.

In terms of data derived from Japanese population, there are several registries including patients with AMI in Japan such as J-MINUET [24, 25], PACIFIC [26], and Tokyo CCU network registry [27]. CVIT has been working on J-PCI registry [28–31], a largest database of patients who underwent PCI in Japan. The current demographics, lesion and procedural characteristics in patients with AMI in Japan can be identified in the J-PCI registry (Tables 3, 4) [from a database including 243436 patients treated in 986 institutions from January 2016 to December 2016].

**Table 3:** Demographics of patients with STEMI and NSTEMI from J-PCI registry

	Overall MI (*n* = 53240)	STEMI (*n* = 41774)	NSTEMI (*n* = 11466)	*P* value (STEMI vs. NSTEMI)
Age (years), mean (SD)	68.78 (12.84)	68.47 (12.93)	69.92 (12.43)	< 0.001
Female	12856 (24.1)	10066 (24.1)	2790 (24.3)	0.609
Cardiogenic shock	6076 (11.5)	5128 (12.4)	948 (8.3)	< 0.001
Risk factors				
Current smoker	20455 (38.4)	16396 (39.2)	4059 (35.4)	< 0.001
Diabetes mellitus	18905 (35.5)	14300 (34.2)	4605 (40.2)	< 0.001
Hypertension	35656 (67.0)	27463 (65.7)	8193 (71.5)	< 0.001
Hypercholesterolemia	30113 (56.6)	23166 (55.5)	6947 (60.6)	< 0.001
History of				
Previous MI	7202 (13.6)	4874 (11.8)	2328 (20.4)	< 0.001
Peripheral vascular disease	1841 (3.5)	1230 (2.9)	611 (5.3)	< 0.001
Previous PCI	9384 (17.7)	6453 (15.5)	2931 (25.6)	< 0.001
Previous CABG	772 (1.5)	418 (1.0)	354 (3.1)	< 0.001
Heart failure	3644 (7.0)	2280 (5.5)	1364 (12.0)	< 0.001
Renal insufficiency	7401 (13.9)	5359 (12.8)	2042 (17.8)	< 0.001
Chronic lung disease (COPD)	1151 (2.2)	859 (2.1)	292 (2.5)	0.002
Door to balloon time				
Min, median (IQR 25th, 75th)	71 (54, 91)	71 (54, 91)	NA	NA
Antiplatelet prescribed before or at procedure				
Dual antiplatelet therapy				
Aspirin + clopidogrel	8085 (19.5)	5749 (18.0)	2336 (24.9)	< 0.001
Aspirin + ticagrelor	29 (0.1)	25 (0.1)	4 (0.0)	0.356
Aspirin + prasugrel	27351 (66.0)	21688 (67.7)	5663 (60.3)	< 0.001
Single antiplatelet therapy	5404 (13.0)	4167 (13.0)	1237 (13.2)	0.719
None	12038 (22.6)	9935 (23.8)	2103 (18.3)	< 0.001
In-hospital mortality	1314 (2.5)	1090 (2.6)	224 (2.0)	< 0.001

Data are counts (percentage) unless otherwise specified

*CABG* coronary artery bypass grafting, *IQR* interquartile range, *MI* myocardial infarction, *NSTEMI* non ST-elevation myocardial infarction, *PCI* percutaneous coronary intervention, *STEMI* ST-elevation myocardial infarction

**Table 4:** Lesion and procedural characteristics in STEMI and NSTEMI from J-PCI registry

	Overall (*n* = 53240)	STEMI (*n* = 41774)	NSTEMI (*n* = 11466)	*P* value (STEMI vs. NSTEMI)
Lesion characteristics				
Lesion location				
LAD/left main	27993 (52.6)	22427 (53.7)	5566 (48.5)	< 0.001
LCX	10730 (20.2)	6642 (15.9)	4088 (35.7)	< 0.001
RCA	20390 (38.3)	16910 (40.5)	3480 (30.4)	< 0.001
Bypass graft	170 (0.3)	76 (0.2)	94 (0.8)	< 0.001
Restenotic lesion	2253 (4.2)	1573 (3.8)	680 (5.9)	< 0.001
Procedure details				
Approach				< 0.001
Transfemoral	21241 (39.9)	17613 (42.2)	3628 (31.6)	
Transradial	30380 (57.1)	22972 (55.0)	7408 (64.6)	
Others (e.g. brachial)	1619 (3.0)	1189 (2.8)	430 (3.8)	
Thrombus aspiration	25579 (48.0)	22626 (54.2)	2953 (25.8)	< 0.001
Distal protection	3874 (7.3)	3386 (8.1)	488 (4.3)	< 0.001
Stent characteristics				
Type of stent				
DES	45622 (85.7)	35962 (86.1)	9660 (84.2)	< 0.001
BMS	1856 (3.5)	1548 (3.7)	308 (2.7)	< 0.001
Other stent (Scaffold)	62 (0.1)	47 (0.1)	15 (0.1)	0.723
No stent used	5876 (11.0)	4352 (10.4)	1524 (13.3)	< 0.001
TIMI flow post-procedure				
Flow 3	52122 (97.9)	40969 (98.1)	11153 (97.3)	< 0.001

Data are counts (percentage)

*BMS* bare metal stent, *DES* drug-eluting stent, *LAD* left anterior descending artery, *LCx* left circumflex artery, *MI* myocardial infarction, *NSTEMI* non ST-elevation myocardial infarction, *RCA* right coronary artery, *STEMI* ST-elevation myocardial infarction, *TIMI* thrombolysis in myocardial infarction

## Primary PCI in STEMI, early invasive vs. conservative strategy in NSTEMI

In ST segment elevation myocardial infarction, primary PCI has been shown to contribute high revascularization success rate, less cardiac events, earlier discharge, even effective in patients with cardiogenic shock [1–19] and consistently recommended by European [20], American [32], and Japanese guidelines.

Regarding non-ST-segment elevation acute coronary syndrome (NSTE-ACS), meta-analysis, based on individual patient data from three studies that compared a routine invasive against a selective invasive strategy, revealed lower rates of death and myocardial infarction at 5-year follow-up in the routine invasive strategy (HR 0.81; 95% CI 0.71–0.93; *P* = 0.002), with the most pronounced difference in high-risk patients [33]. Age, diabetes, previous myocardial infarction, ST-segment depression, hypertension, body mass index (< 25 or > 35 kg/m^2^), and treatment strategy were found to be independent predictors of death and myocardial infarction during follow-up. The results supported a routine invasive strategy but highlight the importance of risk stratification in the decision-making process management as is in the present guidelines.

However, in the ICTUS trial, in which 1200 patients with NSTE-ACS and an elevated cardiac troponin T were randomized to an early invasive strategy versus a selective invasive strategy, 10-year death or spontaneous MI was not statistically different between the 2 groups (33.8 vs. 29.0%, HR 1.12; 95% CI 0.97–1.46; *P* = 0.11). In addition, the 15-year follow-up of the FRISC-II study showed a significant 18-month postponement of the occurrence of death or next MI and 37 months postponement of re-hospitalisation for ischemic heart disease in the early invasive strategy but similar mortality with either strategy [34]. Although the long-term benefit of an early invasive strategy is unclear, the timing of angiography and revascularization should be based on patient risk profile, considering the significant difference between early and delayed strategies in short-term outcome.

Recently, GRACE risk score was applied to the patients with ACS in the Tokyo CCU (cardiovascular care unit) Network Database. A total of 9460 patients with ACS hospitalized at 67 Tokyo CCUs were retrospectively reviewed and there was a strong correlation between the GRACE risk score and in-hospital mortality for patients with STEMI or NSTEMI (*r* = 0.99, *P* < 0.001); however, the correlation was not significant for patients with unstable angina (*r* = 0.35, *P* = 0.126). We recommend use of GRACE score to identify high-risk patients with acute myocardial infarction [35].

### Recommendations

- Primary PCI of the infarct-related artery (IRA) is indicated in STEMI.

#### In case of NSTEMI

- Urgent coronary angiography (< 2 h) is recommended in patients at very high ischemic risk (refractory angina, with associated heart failure, cardiogenic shock, life-threatening ventricular arrhythmias, or hemodynamic instability).
- An early invasive strategy (< 24 h) is recommended in patients with at least one primary high-risk criterion (Table 5).

**Table 5:** Criteria for high risk with indication for invasive management [20]

Primary criteria
1. Relevant rise or fall in troponin
2. Dynamic ST- or T-wave changes (symptomatic or silent)
3. GRACE score > 140
Secondary criteria
4. Diabetes mellitus
5. Renal insufficiency (eGFR < 60 ml/min/1.73 m^2^)
6. Reduced LV function (ejection fraction < 40%)
7. Early post-infarction angina
8. Recent PCI
9. Prior CABG
10. Intermediate to high GRACE risk score (http://www.gracescore.org)

*CABG* coronary artery bypass grafting, *eGFR* estimated glomerular filtration rate, *GRACE* Global Registry of Acute Coronary Events, *LV* left ventricular, *PCI* percutaneous coronary intervention
- An invasive strategy (< 72 h after first presentation) is indicated in patients with at least one high-risk criterion (Table 5) or recurrent symptoms.
- Non-invasive documentation of inducible ischemia is recommended in low-risk patients without recurrent symptoms before deciding on invasive evaluation.

## Practical recommendation for primary percutaneous coronary intervention

### Loading dose DAPT

Prasugrel and ticagrelor reduce ischemic events and mortality in ACS patients compared to clopidogrel and are recommended by current guidelines [20, 36].

In TRITON-TIMI 38, 13608 patients with acute coronary syndromes with scheduled percutaneous coronary intervention were randomized to either prasugrel or clopidogrel. Prasugrel therapy was associated with significantly reduced rates of ischemic events, including stent thrombosis, but with an increased risk of major bleeding, including fatal bleeding. Overall mortality did not differ significantly between treatment groups [36]. In Japanese population, the PRASFIT-ACS study was conducted to confirm the efficacy and safety of prasugrel at loading/maintenance doses of 20/3.75 mg [37]. Japanese patients (*n* = 1363) with acute coronary syndrome undergoing percutaneous coronary intervention were randomized to either prasugrel (20 mg for loading/3.75 mg for maintenance) or clopidogrel (300 mg for loading/75 mg for maintenance). The incidence of MACE at 24 weeks was 9.4% in the prasugrel group and 11.8% in the clopidogrel group (risk reduction 23%, hazard ratio 0.77, 95% confidence interval 0.56–1.07). The incidence of non-coronary artery bypass graft-related major bleeding was similar in both groups (1.9 vs. 2.2%). The results were similar to TRITON-TIMI 38 with a low risk of clinically serious bleeding in Japanese ACS patients.

Regarding ticagrelor, clinical outcomes in a large real-world post-ACS population was studied in a Swedish prospective cohort study in 45073 ACS patients who were discharged on ticagrelor (*N* = 11954) or clopidogrel (*N* = 33119) [38]. The risk of the primary outcome (i.e. composite of all-cause death, re-admission with Ml or stroke) with ticagrelor vs. clopidogrel was 11.7 vs. 22.3% [adjusted HR (HR) 0.85 (95% CI 0.78–0.93)], risk of death 5.8 vs. 12.9% [adjusted HR 0.83 (0.75–0.921)], and risk of Ml 6.1 vs. 10.8% [adjusted HR 0.89 (0.78–1.011)] at 24 months. Re-admission for bleeding with ticagrelor versus clopidogrel was similar. Ticagrelor versus clopidogrel post-ACS was associated with a lower risk of death, Ml, or stroke, as well as death alone. Risk of bleeding was higher with ticagrelor [38]. These real-world outcomes are consistent with the results of the landmark Platelet Inhibition and Patient Outcomes (PLATO) trial [39].

Both prasugrel and ticagrelor are available for clinical use in Japan as well. While the recommended dose of prasugrel is the same as in Europe and United States of America, the Japanese dose of prasugrel was reduced according to the PLASFIT-ACS study [37] (EU: 60 mg loading dose and 10 mg maintenance dose once daily; Japan: 20 mg loading dose and 3.75 mg maintenance dose once daily) (Table 1).

#### Recommendations

- A potent P2Y12 inhibitor (prasugrel or ticagrelor), or clopidogrel if these are not available or are contraindicated, is recommended before (or at latest at the time of) PCI and maintained over 12 months, unless there are contraindications such as excessive risk of bleeding.
- Recommended dose of prasugrel: 20 mg loading dose and 3.75 mg maintenance dose once daily per os (p.o.).
- Recommended dose of ticagrelor: 180 mg p.o. loading dose and 90 mg maintenance dose twice daily.

### Anticoagulation during PCI

According to the 2017 ESC STEMI Guidelines, routine use of unfractionated heparin (UFH) is recommended as a Class I recommendation and routine use of enoxaparin or bivalirudin during primary PCI is a Class IIa recommendation [20].

There has been no placebo-controlled trial evaluating UFH in primary PCI, but there is a large body of experience with this agent. Dosage should follow standard recommendations for PCI (i.e. initial bolus 70–100 U/kg). There are no robust data recommending the use of activated clotting time to tailor dose or monitor UFH, and if activated clotting time is used, it should not delay recanalization of the IRA.

An i.v. bolus of enoxaparin 0.5 mg/kg was compared with UFH in the ATOLL randomized trial including 910 STEMI patients [40]. The primary composite endpoint of 30 day death, MI, procedural failure, or major bleeding was not significantly reduced by enoxaparin (17% relative risk reduction, *P* = 0.063), but there was a reduction in the composite main secondary endpoint of death, recurrent MI or ACS, or urgent revascularization. Importantly, there was no evidence of increased bleeding following the use of enoxaparin over UFH. In a meta-analysis of 23 PCI trials (30966 patients, 33% primary PCI), enoxaparin was associated with a significant reduction in death compared to UFH. This effect was particularly significant in the primary PCI context and was associated with a reduction in major bleeding [41]. In Japan, enoxaparin is approved only for subcutaneous and is practically difficult to use during PCI.

A meta-analysis comparing bivalirudin with unfractionated heparin (UFH) with or without planned use of GP IIb/IIIa inhibitors in patients with STEMI showed no mortality advantage with bivalirudin and a reduction in the risk of major bleeding, but at the cost of an increased risk of acute stent thrombosis [42]. In the recent MATRIX trial including 7213 ACS patients (56% with STEMI), bivalirudin did not reduce the incidence of the primary endpoint (composite of death, MI, or stroke) compared to UFH. Bivalirudin was associated with lower total and cardiovascular mortality, lower bleeding, and more definite stent thrombosis [43]. A post hoc analysis suggested that prolonging bivalirudin with a full-PCI dose after PCI was associated with the lowest risk of ischemic and bleeding events, which is in accordance with the current label of the drug [43]. Bivalirudin could be considered in STEMI, especially in patients at high bleeding risk [44–46]. Bivalirudin is recommended for patients with heparin-induced thrombocytopenia.

After the publication of the 2017 ESC guidelines, the VALIDATE-SWEDEHEART (Bivalirudin versus Heparin in ST-Segment and Non-ST-Segment Elevation Myocardial Infarction in Patients on Modern Antiplatelet Therapy in the Swedish Web System for Enhancement and Development of Evidence-based Care in Heart Disease Evaluated according to Recommended Therapies Registry Trial) multicenter, randomized, registry-based trial was published [47]. Patients with either ST-segment elevation Ml (*N* = 3005) or non ST-segment elevation Ml (*N* = 3001) undergoing PCI and receiving a potent P2Y12 inhibitor (ticagrelor, prasugrel, or cangrelor) without the planned use of glycoprotein IIb/IIIa inhibitors were randomly assigned to receive bivalirudin or heparin during PCI performed predominantly with the use of radial artery access. The primary composite end point (death from any cause, Ml, or major bleeding during 180 days of follow-up) occurred in 12.3% of the patients in the bivalirudin group and in 12.8% in the heparin group (HR 0.96; 95% CI 0.83–1.10; *P* = 0.54). The results were consistent between patients with ST-segment elevation Ml and those with non ST-segment elevation Ml and across other major subgroups. There was no difference between groups in Ml, major bleeding, definite stent thrombosis or mortality. This study shows overall clinical non-inferiority for use of bivalirudin or heparin during PCI for ACS, along with increased cost with use of bivalirudin. Consistently with these findings, the current uptake of bivalirudin in Europe is very low. Bivalirudin remains unavailable in Japan with no evaluation by clinical trials.

Glycoprotein (GP) IIb/IIIa inhibitors are the strongest antiplatelet agents currently available in Europe and in the US, but are not available in Japan. There are three different compounds, namely abciximab, tirofiban, and eptifibatide. However, procedural use of abciximab plus unfractionated heparin (UFH) showed no benefit compared to bivalirudin [44]. In Japan, JEPPORT randomized, placebo-controlled trial (*n* = 973) did not show efficacy of abciximab in reducing the primary endpoint (30-day post-PCI coronary events: death, MI or urgent revascularization) [48]. However, using GP IIb/IIIa inhibitors as bailout therapy in the event of angiographic evidence of a large thrombus, slow- or no-reflow, and other thrombotic complications is reasonable, as recommended in 2017 ESC guidelines [20], although this strategy has not been tested in a randomized trial. Overall, there is no evidence to recommend the routine use of GP IIb/IIIa inhibitors for primary PCI.

#### Recommendations

- Anticoagulation is recommended for all patients in addition to antiplatelet therapy during primary PCI.
- Routine use of unfractionated heparin (UFH) is recommended.

### Approach (femoral vs. radial)

Over recent years, several studies have provided robust evidence in favor of the radial approach as the default access site in ACS patients undergoing primary PCI by experienced radial operators [49, 50]. In the Minimizing Adverse Hemorrhagic Events by TRansradial Access Site and Systemic Implementation of angioX (MATRIX) programme patients were randomized to radial or femoral access, stratified by STEMI (2001 radial, 2009 femoral) and NSTE-ACS (2196 radial, 2198 femoral). MACE occurred in 121 (6.1%) STEMI patients with radial access vs. 126 (6.3%) patients with femoral access [rate ratio (RR) 0.96, 95% CI 0.75–1.24; *P* = 0.76] and in 248 (11.3%) NSTE-ACS patients with radial access vs. 303 (13.9%) with femoral access (RR 0.80, 95% CI 0.67–0.96; *P* = 0.016) (*P*_int_ = 0.25). NACE occurred in 142 (7.2%) STEMI patients with radial access and in 165 (8.3%) patients with femoral access (RR 0.86, 95% CI 0.68–1.08; *P* = 0.18) and in 268 (12.2%) NSTE-ACS patients with radial access compared with 321 (14.7%) with femoral access (RR 0.82, 95% CI 0.69–0.97; *P* = 0.023) (*P*_int_ = 0.76). All-cause mortality and access site-actionable bleeding favored radial access irrespective of ACS type (*P*_interaction_ = 0.11 and *P*_interaction_ = 0.36, respectively) [51]. Radial as compared with femoral access was shown to have consistent benefit across the whole spectrum of patients with ACS, resulting in upgrading recommendation as Class I in 2017 ESC guidelines.

In Japan, the TEMPURA trial randomized patients with AMI undergoing primary PCI to transradial coronary intervention (TRI) group (*n* = 77) and transfemoral coronary intervention (TFI) group (*n* = 72) [52]. The success rate of reperfusion and the incidence of in-hospital MACE were similar in both groups (96.1 and 5.2 vs. 97.1 and 8.3% in TRI and TFI groups, respectively). In a sub-study of PRASFIT-ACS including ACS patients with prasugrel, rates of periprocedural bleeding, bleeding not related to CABG, and puncture site bleeding were consistently lower in the TRI group than in the TFI group [53]. More recently, in a report from the CREDO-Kyoto AMI registry was published [54]. 3662 STEMI patients who had primary PCI by TRI (*N* = 471) or TFI (*N* = 3191) were analyzed. The prevalence of hemodynamically compromised patients (Killip II–IV) was significantly less in TRI group than in TFI group (19 vs. 25%, *P* = 0.002). Cumulative 5-year incidences of death/MI/stroke, and major bleeding were not significantly different between the TRI and TFI groups (26.7 vs. 25.9%, log-rank *P* = 0.91, and 11.3 vs. 11.5%, log-rank *P* = 0.71, respectively). After adjustment for confounders, the risks of the TRI or TFI group were not significant for both death/MI/stroke [hazard ratio (HR) 1.15, 95% confidence interval (CI) 0.83–1.59, *P* = 0.41] and major bleeding (HR 1.29, 95% CI 0.77–2.15, *P* = 0.34), leading to the conclusion that clinical outcomes of transradial approach were not different from those of transfemoral approach in primary PCI for STEMI in the real-world practice.

#### Recommendations

- Radial access is recommended over femoral access if performed by an experienced radial operator.

### Thrombus aspiration

In the new guidelines released by the European Society of Cardiology in 2017 on the management of patients with ST-segment elevation Ml, routine thrombus aspiration was downgraded from IIa to III.

A pooled analysis of individual patient data from three large randomized trials [Thrombus Aspiration During Percutaneous Coronary Intervention in Acute Myocardial Infarction (TAPAS), Thrombus Aspiration in ST-Elevation Myocardial Infarction in Scandinavia (TASTE), and Trial of Routine Aspiration Thrombectomy with PCI Versus PCI Alone in Patients with STEMI (TOTAL)] provided novel insights about thrombus aspiration for ST-elevation MI [55]. By including 18306 patients, the study did not show a significant reduction in cardiovascular death when thrombus aspiration was compared with standard therapy. There were also no differences between thrombus aspiration and no thrombus aspiration with respect to stroke or transient ischemic attack, recurrent Ml, stent thrombosis, heart failure or target vessel revascularization [56]. Although routine use of mechanical thrombus aspiration is no longer recommended, prior safety concerns regarding the risk of stroke could not be confirmed. Because a trend toward reduced cardiovascular death and increased stroke or transient ischemic attack was found in the subgroup of patients with high thrombus burden, future studies may want to investigate improved thrombus aspiration technologies in this high-risk subgroup.

In contrast to the studies mentioned above, earlier studies have shown the benefit of thrombus aspiration in primary PCI [57, 58].

#### Evidence from Japan

There are several studies in Japan showing the benefit of thrombus aspiration in primary PCI.

In the VAMPIRE study [59], randomizing patients with STEMI to primary PCI with (*n* = 180) or without (*n* = 175) upfront thrombus aspiration. There was a trend toward lower incidence of slow or no reflow (primary end point-defined as a thrombolysis in myocardial infarction flow grade < 3) in patients treated with aspiration versus conventional primary PCI (12.4 vs. 19.4%, *P* = 0.07). Rate of myocardial blush grade 3 was higher in the aspiration group (46.0 vs. 20.5%, *P* < 0.001). Aspiration was most effective in patients presenting after 6 h of symptoms onset (slow flow rate: 8.1 vs. 37.6%, *P* = 0.01). Patients presenting late after STEMI appear to benefit the most from thrombectomy.

In an observational study (*n* = 3913) by Nakatani et al. [60], thrombus aspiration was associated with a lower 30-day mortality rate in selected patients with high TIMI risk scores, an age > or = 70 years, diabetes mellitus, or stenting adjustment for baseline characteristics.

In the latest guidelines of Japanese Circulation Society, thrombus aspiration in primary PCI was recommended as Class IIa with level of evidence B. Accordingly, thrombus aspiration is performed frequently in primary PCI in Japan. A comparison of specifications of aspiration device is tabulated in Table 6. From a practical view point, aspiration performance, trackability, and pushability are of importance when choosing an aspiration catheter [61].

**Table 6:** Thrombus aspiration catheters commercially available in Japan

Company	Product name	Guiding catheter compatibility (Fr)	Guidewire compatibility (inch)	Catheter length (cm)	Wire lumen length (mm)	Distal outer diameter (mm)	Distal inner diameter (mm)	Proximal outer diameter (mm)	Proximal inner diameter (mm)	Length of hydrophilic coating (cm)	Shape of aspiration lumen	Stylet
Terumo	Eliminate + SL	6	0.014	140	90	1.70	0.98	1.40	1.05	40	Circle	No
	Eliminate + XL	6	0.014	140	90	1.75	1.10	1.40	1.15	40	Circle	Yes
		7	0.014	140	90	1.98	1.30	1.60	1.35	40	Circle	Yes
Medtronic	Export advance	6	0.014	140	200	1.70	1.09	1.37	1.12	38	Circle	Yes
Kaneka	Thrombuster II	6	0.014	140	10	1.30	1.00	1.30	1.10	30	Circle	Yes
		7	0.014	140	10	1.50	1.20	1.53	1.32	30	Circle	Yes
		8	0.014	140	10	1.73	1.35	1.73	1.50	30	Circle	Yes
		9	0.014	140	10	2.00	1.50	2.00	1.75	30	Circle	Yes
	Thrombuster III SLa	6	0.014	140	120	1.35	1.00	1.35	1.00	30	Circle	No
		7	0.014	140	120	1.55	1.25	1.55	1.25	30	Circle	No
	Thrombuster III GRa	6	0.014	140	120	1.35	1.16	1.35	1.16	30	Circle	Yes
		7	0.014	140	120	1.55	1.36	1.55	1.36	30	Circle	Yes
Nipro	TVAC II	6	0.014	140	240	1.77	0.95	1.40	0.95	24	Circle	Yes^a^
		7	0.014	140	240	1.90	1.18	1.60	1.18	24	Circle	Yes^a^
	TVAC SOFT	6	0.014	135	250	1.50	NA	1.30	NA	25	Crescent	No
		7	0.014	135	250	1.50	NA	1.50	NA	25	Crescent	No
		8	0.014	135	250	1.80	NA	1.80	NA	25	Crescent	No
Goodman	Rebirth Pro 2	6	0.014	136	220	1.35 × 1.62	1.09	1.38	1.11	60	Circle	Yes
		7	0.014	136	220	1.60 × 1.90	1.34	1.58	1.25	60	Circle	Yes

^a^There is TVAC II with or without stylet

Anzai et al. reported that thrombus aspiration facilitates direct stenting without increasing the cost of treatment [62]. Thrombus aspiration can be considered followed by direct stenting, which will be discussed later.

#### Recommendations

- Thrombus aspiration can be considered in primary PCI in absence of GP IIb/IIIa inhibitors.

### Distal protection

The benefit of distal protection using filter device or occlusion balloon has not been confirmed [63, 64]. However, the use of distal protection devices can be considered when plaque burden is large and there is a high possibility of distal embolism or no reflow.

#### Evidences from Japan

Isshiki et al. reported initial clinical experience with Filtrap distal protection filter [65]. Filtrap was successfully delivered and deployed distal to the lesion in 13 of 14 patients (93%). Embolic debris was entrapped in 8 (62%) of these cases. All patients were free from in-hospital events except for one patient with a large anterior acute myocardial infarction who received an emergency surgery due to a free wall cardiac rupture. In the ASPARAGUS trial (*n* = 341), patients with AMI were randomized to either stenting with or without GuardWire Plus [66]. The rates of slow flow and no-reflow immediately after PCI were 5.3 and 11.4% in the GuardWire Plus and control groups, respectively (*P* = 0.05). Blush score 3 acquisition rates immediately after PCI were 25.2 and 20.3% in the GuardWire Plus and control groups, respectively (*P* = 0.26), and the rates at 30 days after PCI were 42.9 and 30.4%, respectively (*P* = 0.035). In the CANARY pilot trial, near-infrared spectroscopy and intravascular ultrasound were performed at baseline, and lesions with a maximal lipid core burden index over any 4-mm length (maxLCBI_4mm_) ≥ 600 were randomized to PCI with versus without a distal protection filter. Among 31 randomized lesions with maxLCBI_4mm_ ≥ 600, there was no difference in the rates of periprocedural MI with versus without the use of a distal protection filter (35.7 vs. 23.5%, *P* = 0.69). More recently, the VAMPIRE 3 trial randomized 200 ACS patients who had attenuated plaque with a longitudinal length of ≥ 5 mm by pre-PCI intravascular ultrasound to either distal protection (DP) by filter or conventional treatment (CT) [67]. The primary endpoint of no-reflow phenomenon occurred in 26.5% of the DP group (*n* = 98) and 41.7% of the CT group (*n* = 96; *P* = 0.0261) and the corrected TIMI frame count after revascularization was significantly lower in the DP group (23 vs 30.5; *P* = 0.0003). In addition, the incidence of in-hospital adverse cardiac events was significantly lower in the DP group than in the CT group (0 vs 5.2%; *P* = 0.028). Future studies may further elucidate whether distal protection is beneficial in selected patient.

In contrast, distal embolic protection during PCI of saphenous vein graft is confirmed in a multicenter randomized controlled trial. In the SAFER randomized trial, a composite of death, myocardial infarction, emergency bypass, or target lesion revascularization by 30 days was observed in 16.5% in the control group and 9.6% in the embolic protection device (*P* = 0.004). This 42% relative reduction in major adverse cardiac events was driven by myocardial infarction (8.6 versus 14.7%, *P* = 0.008) and “no-reflow” phenomenon (3 versus 9%, *P* = 0.02). Clinical benefit was seen even when platelet glycoprotein IIb/IIIa receptor blockers were administered (61% of patients), with composite end points occurring in 10.7% of protection device patients versus 19.4% of control patients (*P* = 0.008). This study demonstrated the importance of prevention of distal embolization in saphenous vein graft. Currently available filter devices in Japan are tabulated in Table 7.

**Table 7:** Filter devices for distal protection commercially available in Japan

Company	Product name	Filter diameter at expansion (mm)	Guidewire compatibility (inch)	Length (cm)
Nipro	Filtrap	3.5	0.014	180
		5	0.014	180
		6.5	0.014	180
		6.5	0.014	300
		8	0.014	180
		8	0.014	300
Tri-Med	Parachute	5	0.014	190
		5	0.014	270
		6.5	0.014	190
		6.5	0.014	270
		8	0.014	270
		8	0.014	50
		8	0.014	190

#### Recommendations

- Distal protection can be considered in selective cases when plaque burden is large and there is a high possibility of distal embolism or no reflow or cases with myocardial infarction in saphenous vein graft.

### Pharmacological intervention for no reflow

In 2017 ESC guidelines [20], using GP IIb/IIIa inhibitors as bailout therapy is considered as Class IIa in the event of angiographic evidence of a large thrombus, slow- or no-reflow, although this strategy has not been tested in a randomized trial.

#### Evidences from Japan

Miyazawa et al. [68] studied the effect of nicorandil in STEMI, randomizing patients with STEMI to nicorandil group (*n* = 35) or control group (*n* = 35). In nicorandil group, 2 mg of nicorandil was injected directly into the infarct area prior to reperfusion by PCI. With nicorandil infusion, additional ST elevations without chest pain were observed for a few minutes in 94% of cases. However, no ventricular fibrillation or ventricular tachycardia occurred. TIMI myocardial perfusion grade 3 was significantly higher in nicorandil group (40 vs. 17%, *P* < 0.01). Rates of adverse events were similar, however, left ventricular regional wall motion score significantly improved in nicorandil group (*P* < 0.05). The effect of nicorandil was pronounced in patients without ischemic preconditioning.

Kobatake et al. compared the effects of nitroprusside (*n* = 25) with nicorandil (*n* = 24) on the slow/no-reflow phenomenon during primary PCI [69]. The degree of improvement in TIMI flow grade (post–pre/pre) and TIMI frame count (pre–post/pre) showed that nitroprusside was more effective than nicorandil (nitroprusside vs. nicorandil: 0.88 ± 0.79, 0.37 ± 0.37, *P* = 0.008; 0.59 ± 0.23, 0.36 ± 0.27, *P* = 0.003, respectively). At 1 year, rate of MACE was not significantly different (5/25 vs. 9/24, *P* = 0.175).

More recently, a network meta-analysis was published comparing the effect of 7 intracoronary agents (adenosine, anisodamine, diltiazem, nicorandil, nitroprusside, urapidil, and verapamil) on the no-reflow phenomenon in patients with STEMI undergoing primary PCI, including 41 randomized control trials with 4069 patients [70]. Anisodamine (α1 adrenergic receptor antagonist used in the treatment of acute circulatory shock in China) was associated with improved post-procedural TIMI flow grade, more occurrences of ST-segment resolution, and improvement of LVEF. The cardioprotective effect of anisodamine conferred a MACE-free survival benefit. Additionally, nitroprusside was regarded as efficient in improving coronary flow and clinical outcomes. Compared with standard care, adenosine, nicorandil, and verapamil improved coronary flow but had no corresponding benefits regarding cardiac function and clinical outcomes.

Considering GP IIb/IIIa inhibitors and anisodamine are not available in Japan, use of nicorandil or nitroprusside prior to reperfusion by primary PCI can be considered reasonable.

#### Recommendations

- Intracoronary injection of nicorandil can be considered to bail out in case of slow flow or no-reflow.

### Direct stenting

Evidence in favor of direct stenting (stenting without predilation) in patients with STEMI comes from several studies [71]. Loubeyre et al. [72] randomized 206 patients with STEMI to direct stenting or stent implantation after balloon predilation. The composite angiographic [corrected thrombolysis in myocardial infarction (TIMI) frame count, slow-flow/no-reflow or distal embolization] endpoint (11.7 vs. 26.9%; *P* = 0.01) and ST-segment resolution (79.8 vs. 61.9%; *P* = 0.01) were better among patients randomized to direct stenting than among those randomized to stent implantation after predilation [72]. In the Harmonizing Outcomes with Revascularization and Stents in Acute Myocardial Infarction (HORIZONS-AMI), direct stenting (*n* = 698) compared with conventional stenting after predilation (*n* = 1830) was associated with better ST-segment resolution at 60 min after the procedure (median: 74.8 vs. 68.9%; *P* = 0.01) and lower 1-year rates of all-cause mortality (1.6 vs. 3.8%; *P* = 0.01) and stroke (0.3 vs. 1.1%; *P* = 0.049) [73]. The EUROTRANSFER Registry that included 1419 patients showed that direct stenting (*n* = 276) was superior to stenting after predilation in terms of post-procedural TIMI flow grade of 3 (94.9 vs. 91.5%; *P* = 0.02), no-reflow (1.4 vs. 3.4%; *P* = 0.035), ST-segment resolution of > 50% (86.2 vs. 76.3%; *P* = 0.016) and 1-year mortality (2.9 vs. 6.5%; *P* = 0.047 after adjustment for propensity score) [74]. Direct stenting may be advantageous over stenting after predilation in several aspects including the use of fewer and shorter stents, shorter fluoroscopy time and less use of contrast media and reduced microvascular dysfunction/obstruction and no-reflow by reduced distal embolization. Potential disadvantages of direct stenting may include: failure to reach and/or to cross the lesion, stent loss, erroneous estimation of stent length, difficulty with stent positioning (especially in case of persistent TIMI flow 0–1), underexpansion of the stent in an undilatable (i.e., calcified) lesion and stent undersizing due to underestimation of vessel diameter because of reduced flow [75]. Notwithstanding these disadvantages, direct stenting is considered almost as a default strategy during primary PCI.

#### Recommendations

- Direct stenting is recommended in primary PCI.

### Balloon angioplasty

The clinical efficacy of balloon angioplasty for STEMI is limited due to the relatively high percentage of restenosis caused by elastic recoil and late negative remodeling [76]. Several studies showed the need for repeat revascularization was significantly reduced by the use of coronary stents [77–79]. There are also Japanese evidences supporting this fact in patients with AMI [80, 81]. Nonetheless, stent implantation did not result in lower rates of recurrent myocardial infarction (MI) or death, when compared with balloon angioplasty alone. Subsequently, numerous randomized trials demonstrated a further reduction in target lesion revascularization (TLR) could be achieved when using drug-eluting stents (DES) as opposed to bare-metal stents (BMS). Equivalent to studies comparing balloon angioplasty with stenting, though, none of these studies demonstrated a reduction in recurrent MI or death [82–84]. An important limitation of stent usage is a persistent risk of stent thrombosis and/or in-stent restenosis even years after implantation, particularly in patient subsets as STEMI [85–90].

Considering stent implantation may even induce no-reflow and thereby expand infarct size [91–93], it may be reasonable to refrain from stenting if coronary flow is restored and no significant stenosis persists after thrombus aspiration and balloon dilatation. Indeed, recent studies have demonstrated it is safe to defer stent implantation in the acute phase of STEMI [94, 95]. Considering the absence of superiority with regard to hard clinical end points and the potential short- and long-term disadvantages of stent implantation, angioplasty with a drug coated balloon (DCB) without stenting may well serve as a therapeutic strategy of choice in STEMI.

The PAPPA pilot study was the first prospective clinical trial studying the efficacy and safety of a DCB only strategy in primary PCI for STEMI [96]. Additional stenting was allowed only in case of type C–F coronary dissection or residual stenosis > 50%. All patients were treated with i.v. bivalirudin. Of 100 consecutive STEMI patients, 59 patients were treated with a DCB only strategy, whereas bail-out stenting was required in 41 patients. At 1-year, a total of five major adverse cardiac events were reported (5%). Cardiac death was seen in two patients, while three patients underwent TLR. Although in this pilot study the rate of bail-out stenting was relatively high, the use of a DCB angioplasty-only strategy in the setting of primary PCI seems to be a safe and feasible treatment modality. Thus far, no angiographic data are available for the use of a DCB only strategy in STEMI.

In the INNOVATION study, 114 patients receiving primary PCI for STEMI were randomized into deferred stenting (DS) or immediate stenting (IS) [97]. In the DS group, the primary procedures included thrombus aspiration and balloon angioplasty and the second-stage stenting procedure was scheduled to be performed at 3–7 days after primary reperfusion procedure. DS did not significantly reduce infarct size (15.0 versus 19.4%; *P* = 0.112) and the incidence of microvascular obstruction (42.6 versus 57.4%; *P* = 0.196), compared with IS. However, in anterior wall myocardial infarction, infarct size (16.1 versus 22.7%; *P* = 0.017) and the incidence of microvascular obstruction (43.8 versus 70.3%; *P* = 0.047) were significantly reduced in the DS group.

The REVELATION trial plans to randomize 120 patients presenting with STEMI either to treatment with a DCB or DES [98] (NCT02219802). The primary endpoint is non-inferiority of the functional assessment of the infarct-related lesion by FFR at 9 months after initial treatment.

#### Recommendations

- Currently, primary PCI using balloon-only strategy is not recommended over direct stenting.

### Pre-procedural IVUS/OCT

In ESC guideline of myocardial revascularization [99], intravascular imaging is recommended only in case of restenosis and stent thrombosis to detect stent-related mechanical problems and to assess and guide PCI in left main stem (IIa).

#### Identification of culprit lesion

IVUS and OCT detect plaque ruptures in about half of ST-elevation myocardial infarction. However, the superior resolution and obligatory flushing with OCT sharply outlines the rupture cavity and residual fibrous cap fragment to optimize ruptured plaque identification. de Feyter and Ozaki previously demonstrated plaque rupture and thrombus were more frequently found in ACS than those in stable angina by angioscopy, while IVUS failed to discriminate unstable from stable plaque [100]. More recently, Kubo et al. reported, when compared with the gold standard of angioscopy, OCT can identify a thrombus better than IVUS and differentiate between red and white thrombus although red thrombus can shadow and obscure underlying plaque morphology [101].

While pathological studies reported that plaque erosion plays a role in ACS, there was no clear OCT definition of plaque erosion previously. While Ozaki and his colleagues proposed that OCT-derived intact fibrous cap (IFC-ACS) can be plaque erosion for the first time, contrary to ruptured fibrous cap (RFC-ACS), distinct culprit lesion characteristics associated with IFC-ACS mechanisms are not identified by CT angiography or IVUS [102]. OCT has been used to monitor changes in thrombus burden when lesions are treated with thrombus aspiration or with pharmacotherapy. [103, 104].

In addition, combined IVUS–NIRS imaging, in particular where an increased plaque burden and lipid component present, is able to differentiate culprit lesions from non-culprit lesions with a high accuracy in STEMI [105, 106] and NSTEMI [107].

#### Likelihood of distal embolization or periprocedural myocardial infarction during stent implantation

Thin-cap fibroatheromas not only cause plaque rupture and thrombosis but also contribute to myonecrosis during stenting. Findings associated with perimyocardial infarction are greyscale IVUS-attenuated plaques, especially when the amount of attenuated plaque is large and begins closer to the lumen than to the adventitia; when large virtual histology-IVUS necrotic core or a virtual histology-thin-cap fibroatheroma or similar findings with integrated backscatter-IVUS (lipid) or iMap (necrotic core) are present; when an OCT-thincap fibroatheroma is present; when large lipid-rich plaques are detected by OCT or NIRS; or when plaque rupture is detected by IVUS or OCT [108, 109]. However, the positive predictive value is poor and one trial [110] did not show superiority of distal protection when treating lipid-rich plaques. Conversely, the absence of these findings indicates a low probability of a peri-myocardial infarction with a high-negative predictive value.

#### Recommendations

- IVUS and/or OCT should be considered to detect stent-related mechanical problems.
- IVUS can be used to assess severity and optimize treatment of unprotected left main lesions.

### Stent

#### Drug-eluting stents

Several randomized controlled trials of DES versus DES reported long-term follow-up in the past year [111–115]. The overall picture from these comparisons based on non-inferiority trials suggests that the 1 year and long-term outcomes with newer-generation DES is very good without notable differences between brands.

In a DES versus DES comparison with 1-year follow-up available, the sirolimus-eluting, thin-strut biodegradable-polymer Orsiro stent was evaluated in the BIOFLOW V study (*N* = 1334) and compared with the durable-polymer Xience everolimus-eluting stent (EES). Six percent of patients in the Orsiro group and 10% in the Xience group met the 12-month primary endpoint of TLF (*P* = 0.0399). It is noteworthy that the Xience stent in the BIOFLOW V had higher TLF rate in selected low-risk patients at 12-month follow-up than in an “all-comers” population at 2-year follow-up in the previous SORT OUT IV trial (5%) [116]. The difference in TLF was primarily driven by a difference in target-vessel Ml (4.7 vs. 8.3%), which was not explained by differences in definite stent thrombosis (0.5 vs. 0.7%) [117].

In the DESSOLVE III randomized, all-comer trial comparing bioresorbable polymer MiStent sirolimus-eluting stent and durable polymer Xience EES, TLF at 12 months occurred 5.8% in the MiStent group and 6.5% in the Xience group (*P*_non-inferiority_ = 0.0001). The rate of definite or probable stent thrombosis at 12 months was 0.7 and 0.9% with MiStent and Xience, respectively (*P* = 0.76).

The SENIOR trial randomized elderly patients undergoing PCI to DES or BMS with use of a short duration of dual antiplatelet therapy (DAPT for 1 month in elective patients, 6 months in patients with ACS). The study found a significant reduction in all-cause mortality, Ml, stroke, and ischemia-driven target lesion revascularization in the DES group [118]. The conclusion is that BMS should no longer be preferred to new generation DES when high bleeding risk is of concern and shortened duration of DAPT is desired.

In a network meta-analysis in patients with STEMI undergoing primary PCI (12453 patients from 22 trials) [119], CoCr-EES was associated with significantly lower rates of cardiac death or MI and stent thrombosis than BMS. CoCr-EES was also associated with significantly lower rates of 1-year ST than paclitaxel-eluting stents (PES). Sirolimus-eluting stents (SES) were also associated with significantly lower rates of 1-year cardiac death/myocardial infarction than BMS. CoCr-EES, PES, and SES, but not zotarolimus-eluting stents, had significantly lower rates of 1-year target vessel revascularization (TVR) than BMS, with SES also showing lower rates of TVR than PES. Another network meta-analysis with longer follow-up data analyzed twelve trials with 9673 patients [120]. Second generation DES was associated with significantly lower incidence of definite or probable stent thrombosis (OR 0.59, 95% CI 0.39–0.89), MI (OR 0.59, 95% CI 0.39–0.89), and TVR at 3 years (OR 0.50: 95% CI 0.31–0.81) compared with BMS. In addition, there was a significantly lower incidence of MACE with second generation DES versus BMS (OR 0.54, 95% CI 0.34–0.74) at 3 years. Overall, use of second generation DES is encouraged; however, an updated network meta-analysis is awaited to compare increasing varieties of drug-eluting stents.

#### Drug-coated stents

The LEADERS-FREE (Prospective Randomized Comparison of the BioFreedom Biolimus A9 Drug-Coated Stent versus the Gazelle Bare-Metal Stent in Patients at High Bleeding Risk) study compared the polymer-free biolimus-eluting Biofreedom stent with a bare metal stent (BMS) in a cohort (*N* = 2466) at high risk of bleeding. In a subgroup analysis of 659 ACS patients, treatment with the BioFreedom stent remained more effective (clinically driven target-lesion revascularization 3.9 vs. 9.0%, *P* = 0.009) and safer (cumulative incidence of cardiac death, Ml, or definite or probable stent thrombosis 9.3 vs. 18.5%, *P* = 0.001), driven by significantly lower rates of cardiac mortality (3.4 vs. 6.9%, *P* = 0.049) and Ml (6.9 vs 13.8%, *P* = 0.005) [121].

These results confirm the clinical utility of the drug-coated stents for patients at high bleeding risk and a direct comparison with current generation DES would be of great interest.

#### Evidence from Japan

There are scarce randomized studies comparing stents in Japan. Sawada et al. randomized patients with STEMI to receive EES (*n* = 23) or SES (*n* = 12) and compared arterial healing by OCT [122]. Both the EES and SES showed an excellent suppression of neointimal proliferation in the culprit lesion. The frequency of uncovered and malapposed struts of EES was significantly lower than that of SES (2.7 vs. 15.7%, *P* < 0.0001, 0.7 vs. 2.3%, *P* < 0.0001, respectively). EES may promote better arterial healing response than SES in patients with STEMI. In the RESET all-comer trial, patients were assigned to either EES (*n* = 1596) or SES (*n* = 1600) [123]. At 3 years, EES was noninferior to SES on the primary safety end point (all-cause death or myocardial infarction; 10.1 versus 11.5%; noninferiority *P* < 0.001; and superiority *P* = 0.19). Cumulative incidence of definite stent thrombosis was low and was not significantly different between the 2 groups (0.5 versus 0.6%; *P* = 0.81). The NAUSICA trial randomized patients with STEMI to Nobori biolimus A9 eluting stent (BES) or BMS and aimed to compare MACE at 1 year. However, the main result has not yet been published. In the NEXT randomized trial, patients scheduled for PCI using DES were randomized to Nobori biodegradable polymer BES (1617 patients) or Xience durable polymer EES (1618 patients) without any exclusion criteria [124]. At 3 years, the primary safety end point of death or myocardial infarction occurred in 159 patients (9.9%) in the BP-BES group and in 166 patients (10.3%) in the DP-EES group, demonstrating noninferiority of BP-BES relative to DP-EES (*P*_noninferiority_ < 0.0001 and *P*_superiority_ = 0.7).

#### Recommendations

- Stenting with new-generation DES is recommended over BMS for primary PCI.

### Post-procedural IVUS/OCT

Post-procedural IVUS/OCT is used to evaluate stent under-expansion, malapposition, tissue protrusion, dissection, geographic miss and thrombus.

In the IVUS-XPL trial [125], 1400 patients with long lesions were randomized to IVUS versus angiographic guidance. IVUS guidance was associated with a lower MACE rate of 2.9 versus 5.8% (*P* = 0.007). In CLI-OPCI observational study (*n* = 670), OCT guidance was associated with a significantly lower risk of cardiac death or MI as compared to angiographic only guidance [adjusted OR = 0.49 (0.25–0.96), *P* = 0.037]. Intravascular imaging-guided PCI has a potential to reduce cardiac death, major adverse cardiac events, stent-thrombosis, and target lesion revascularization as compared with angiography-guided PCI [126]. OCT-guided PCI is non-inferior to IVUS-guided PCI in terms of stent expansion in the ILUMIEN III trial [127] and clinical outcome in the OPINION trial [128] from Japan.

In general, a small edge dissection found on OCT which is undetected on angiography most likely does not have a clinical impact [129–132]. However, the following factors need to be considered: longitudinal and circumferential extension of dissection, and the depth of dissection (intima, media or even adventitia). In the ILUMIEN III [127], edge dissections were categorized as major if they constituted ≥ 60° of the circumference of the vessel at the site of dissection and/or were ≥ 3 mm in length. In this trial, when the intra-dissection lumen area is < 90% of the respective reference area, additional stent implantation was considered. In CLI-OPCI-II trial [133], dissection was defined on OCT as a linear rim of tissue with a width of ≥ 0.2 mm and a clear separation from the vessel wall or underlying plaque. In this retrospective multicenter registry, the acute dissection in the distal stent edge was an independent predictor for major adverse cardiac events.

If the malapposition distance from the endoluminal lining of strut to the vessel wall is < 250 µm, such struts likely become in contact with vessel wall at follow-up. Therefore, such small malapposition may be less relevant [134, 135]. The clinical relevance of acute malapposition on stent failure is not yet fully established [133, 136–138]. Ozaki et al. reported the fate of stent malapposition with serial (post and 10 months follow-up) OCT examinations [139]. They found that of the 4320 struts in 616 slices in 32 patients with sirolimus eluting stent (SES), persistent malapposition (incomplete stent apposition; ISA) was observed in 4.67%, resolved/healed malapposition was 2.48%, late acquired malapposition was 0.37% and most of them was well apposed with neointimal coverage in 84.89% and without coverage in 7.59% [139]. More interestingly, thrombus was visualised in 20.6% of struts with ISA at follow-up and in 2.0% of struts with good apposition (*P* < 0.001) [139]. The temporal evolution and disappearance of malapposition made the investigation of clinical relevance of strut malapposition more complicated.

#### Recommendations

- IVUS or OCT can be used to optimize stent implantation.
- Acute incomplete stent apposition with a distance of ≤ 250 µm is likely to be resolved at follow-up. Additional post-dilatation is considered when malapposition distance is > 250 µm.
- Most edge dissection detected on OCT is clinically silent, whereas additional stenting may be performed if the width of distal edge dissection is ≥ 200 µm [133].

### Mechanical hemodynamic support

IABP counterpulsation is the most widely used mechanical support for the treatment of cardiogenic shock, based on the beneficial effect of aortic diastolic inflation and rapid systolic deflation, improving myocardial and peripheral perfusion and reducing afterload and myocardial oxygen consumption.

The previous ESC guidelines stated that intra-aortic balloon pumping may be considered in cardiogenic shock after STEMI (IIb) [21]. However, IABP counterpulsation does not improve outcomes in patients with STEMI and cardiogenic shock without mechanical complications [23, 140], nor does it significantly limit infarct size in those with potentially large anterior MIs [22]. The latest ESC guidelines no longer recommend routine IABP counterpulsation in cardiogenic shock except selected patients (i.e. severe mitral insufficiency or ventricular septal defect).

In other countries, mechanical LV assist devices (LVADs), including percutaneous short-term mechanical circulatory support devices (i.e. intra-cardiac axial flow pumps and arterial-venous extracorporeal membrane oxygenation), have been used in patients not responding to standard therapy, including inotropes, fluids, and IABP, but evidence regarding their benefits is limited [141]. A small exploratory trial studying the Impella CP percutaneous circulatory support device did not find any benefit compared with IABP in AMI complicated by cardiogenic shock [142]. Therefore, short-term mechanical circulatory support may be considered as a rescue therapy to stabilize the patients and preserve organ perfusion (oxygenation) as a bridge to recovery of myocardial function, cardiac transplantation, or even LV assist device destination therapy on an individual basis [143, 144].

A structured approach to determine the best adjunctive mechanical circulatory support device requires understanding the mechanisms, technical requirements, and hemodynamic responses of each device [145] (Table 8). Device escalation is often required if the initial support device (usually IABP) does not improve hemodynamics and end organ perfusion. Venoarterial extracorporeal membrane oxygenation (VA-ECMO) is often used in a combination with IABP to reduce the afterload increased by the retrograde flow. In a retrospective cohort study using propensity score matching in the Japanese Diagnosis Procedure Combination national inpatient database [146], all-cause 28-day mortality and in-hospital mortality were significantly lower in the IABP combined with VA-ECMO group than the VA-ECMO-alone group (48.4 vs 58.2%; *P* = 0.001 and 55.9 vs 64.5%; *P* = 0.004, respectively). The proportion of patients weaned from VA-ECMO was significantly higher in the IABP combined with VA-ECMO group than in the VA-ECMO-alone group (82.6 vs 73.4%; *P* < 0.001).

**Table 8:** Comparison of mechanical circulatory support system. Modified from Atkinson et al. [145]

	IABP	IMPELLA	VA-ECMO
Cardiac flow	0.3–0.5 L/min	1–5 L/min (Impella 2.5, Impella CP, Impella 5)	3–7 L/min
Mechanism	Aorta	LV → Ao	RA → Ao
Maximum implant days	Weeks	7 days	Weeks
Sheath size	7–8 Fr	13–14 Fr Impella 5.0–21 Fr	14–16 Fr Arterial 18–21 Fr Venous
Femoral artery size	> 4 mm	Impella 2.5 and CP: 5–5.5 mm Impella 5: 8 mm	8 mm
Cardiac synchrony or stable rhythm	Yes	No	No
Afterload	↓	↓	↑ ↑ ↑
Mean arterial pressure	↑	↑ ↑	↑ ↑
LVEDP	↓	↓ ↓	⟷
PCWP	↓	↓ ↓	⟷
LV preload	–	↓ ↓	↓
Coronary perfusion	↑	↑	–
Myocardial oxygen demand	↓	↓ ↓	⟷

*Ao* aorta, *IABP* intra-aortic balloon pump, *LA* left atrium, *LV* left ventricle, *LVEDP* left ventricular end diastolic pressure, *RA* right atrium, *PCWP* pulmonary capillary wedge pressure, *VA-ECMO* venoarterial extracorporeal membrane oxygenation

There have been several clinical reports suggesting the combined use of Impella with IABP [147, 148]. However, this combination may decrease Impella forward flow during diastole due to diastolic pressure augmentation from the IABP [149].

The latest guidelines for STEMI from Japanese Circulation Society recommended IABP use as Class I with level of evidence B, considering the percutaneous LVADs were not broadly available in Japan. However, the Impella 2.5 and Impella 5.0 heart pumps received Pharmaceuticals and Medical Devices Agency (PMDA) approval from the Japanese Ministry of Health, Labor and Welfare (MHLW) in September 2016 and received reimbursement, effective as of September 2017. Proper selection of patients, institutional criteria are being reviewed in J-PVAD (http://j-pvad.jp).

#### Recommendations

- Routine intra-aortic balloon pumping is not indicated.
- Intra-aortic balloon pumping should be considered in patients with hemodynamic instability/cardiogenic shock due to mechanical complications.
- In patients presenting refractory shock, short-term mechanical support (Impella or ECMO) may be considered.

### DAPT in maintenance phase

#### Risk stratification for bleeding

The PRECISE-DAPT score (age, creatinine clearance, hemoglobin, white-blood-cell count, and previous spontaneous bleeding) was derived from 14963 patients treated with different duration of DAPT (mainly aspirin and clopidogrel) after coronary stenting and showed a c-index for out-of hospital TIMI major or minor bleeding of 0.73 (95% CI 0.61–0.85) [150]. A longer DAPT duration significantly increased bleeding in patients at high risk (score ~ 25), but did not in those with lower bleeding risk profiles, and exerted a significant ischemic benefit only in this latter group. As stated in the new ESC/EACTS Consensus document on DAPT, the use of risk scores such as PRECISE-DAPT designed to evaluate the benefits and risks of different DAPT durations ‘may be considered’ to support decision making [151].

Recently, Yoshikawa et al. reported that, in a pooled cohort of three studies conducted in Japan (12223 patients from the CREDO Kyoto registry cohort-2, RESET and NEXT), the DAPT score successfully stratified ischemic and bleeding risks, although the ischemic event rate was remarkably low even in high DAPT score [152].

#### DAPT duration

**Fig. 1:**
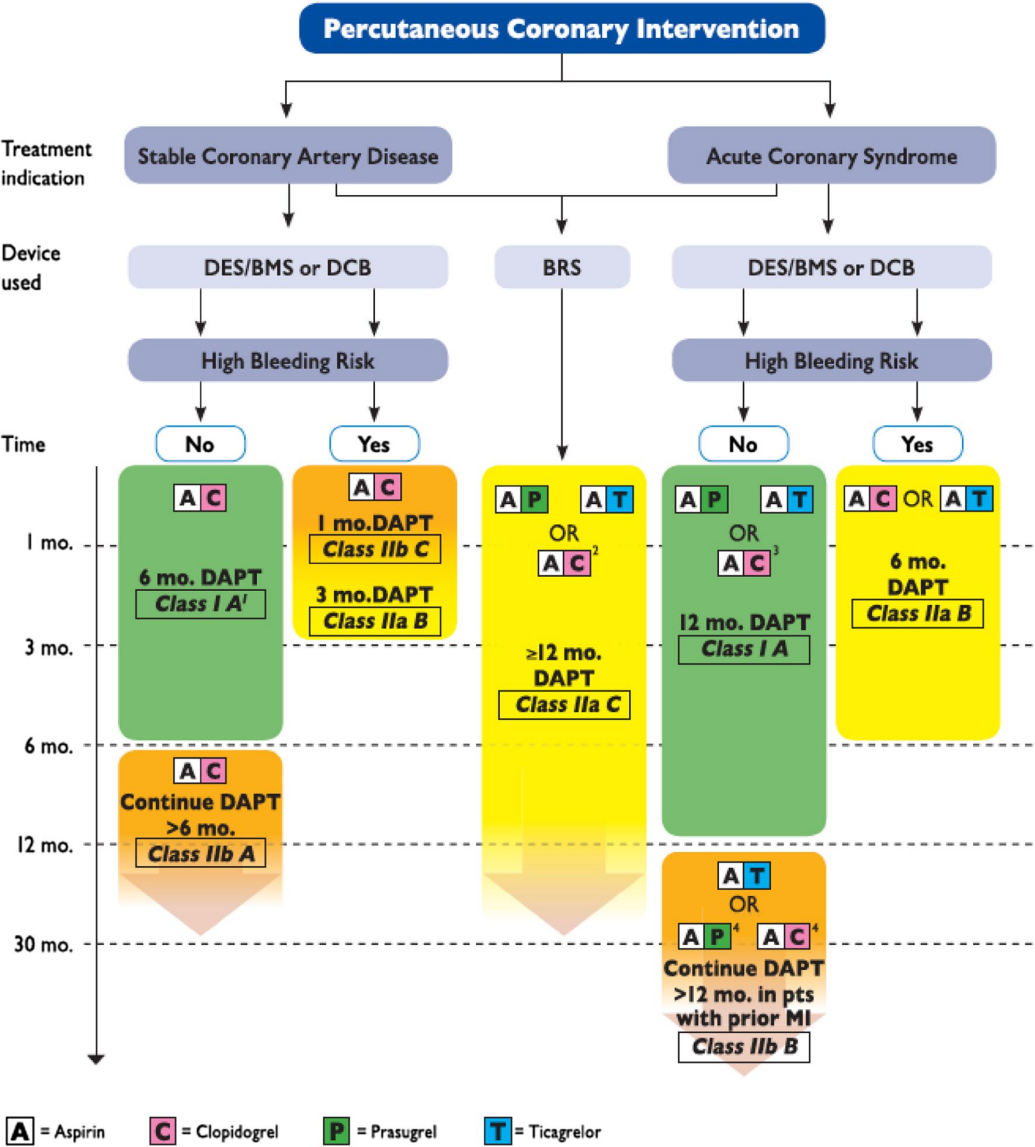
Algorithm for dual antiplatelet therapy (DAPT) in patients treated with percutaneous coronary intervention. High bleeding risk is considered as an increased risk of spontaneous bleeding during DAPT (e.g. PRECISE-DAPT score ≥ 25). Colour-coding refers to the ESC Classes of Recommendations (green = Class I; yellow = IIa; orange = Class IIb). Treatments presented within the same line are sorted in alphabetic order, no preferential recommendation unless clearly stated otherwise. ^1^After PCI with DCB, 6-month DAPT should be considered (Class IIa B). ^2^If patient presents with Stable CAD or, in case of ACS, is not eligible for a treatment with prasugrel or ticagrelor. ^3^If patient is not eligible for a treatment with prasugrel or ticagrelor. ^4^If patient is not eligible for a treatment with ticagrelor. *ACS* acute coronary syndrome, *BMS* bare-metal stent, *BRS* bioresorbable vascular scaffold, *CABG* coronary artery bypass graft surgery, *DCB* drug-coated balloon, *DES*: drug-eluting stent, *PCI* percutaneous coronary intervention, *Stable CAD* stable coronary artery disease. Reproduced with permission from Valgimigli et al. [151]

Recommendations on duration of DAPT in patients with ACS and after elective stenting have been given in the ESC/EACTS focused update on DAPT (Fig. 1) [151]. Recently, the 2 year follow-up report of the Is There a Life for DES After Discontinuation of Clopidogrel (ITALIC) study (*N* = 2031) confirmed the 1-year results and showed that patients receiving 6-month DAPT after PCI with second-generation DES have similar outcomes to those receiving 24-month DAPT [153].

Another study pooled patient-level data from 6 randomized controlled trials and investigated the efficacy and safety of long-term (≥ 12 months) versus short-term (3 or 6 months) DAPT with aspirin and clopidogrel after PCl [154]. Of 9577 patients included in the pooled dataset for whom procedural variables were available, 1680 (17.5%) underwent complex PCI. Overall, 85% of patients received new-generation DES. At a median follow-up time of 392 days, patients who underwent complex PCI had a higher risk of MACE [(HR) 1.98; 95% (CI) 1.50–2.60; *P* < 0.0001]. Compared with short-term DAPT, long-term DAPT yielded significant reductions in MACE in the complex PCI group (adjusted HR 0.56; 95% CI 0.35–0.89) versus the noncomplex PCI group (adjusted HR 1.01; 95% CI 0.75–1.35; *P* for interaction = 0.01). The magnitude of the benefit with long-term DAPT was progressively greater per increase in procedural complexity. Long-term DAPT was associated with increased risk for major bleeding, which was similar between groups [154]. Results were consistent by per-treatment landmark analysis and further establish procedural complexity is an important parameter to take into account in tailoring upfront duration of DAPT [151].

A large individual patient data pairwise and network meta-analysis comparing short-term (≤ 6 months) versus long-term (1-year) DAPT as well as 3 versus 6-month versus 1-year DAPT included 11473 patients [155]. The primary study outcome was the 1-year composite risk of Ml or definite/probable stent thrombosis. Six trials including 11473 randomized patients in which DAPT after DES consisted of aspirin and clopidogrel: 6714 (58.5%) had stable CAD and 4758 (41.5%) presented with ACS, the majority of whom (67.0%) had unstable angina. In ACS patients, ≤ 6-month DAPT was associated with non-significantly higher 1-year rates of Ml or stent thrombosis compared with 1-year DAPT (HR 1.48, 95% CI 0.98–2.22), whereas in stable patients, the rates of Ml and stent thrombosis were similar between the two DAPT strategies (HR 0.93, 95% CI 0.65–1.35). By network meta-analysis, 3-month DAPT, but not 6-month DAPT, was associated with higher rates of Ml or stent thrombosis in ACS, whereas no significant differences were apparent in stable patients. Short DAPT was associated with lower rates of major bleeding compared with 1-year DAPT, irrespective of clinical presentation. All-cause mortality was not significantly different with short versus long DAPT in both patients with stable CAD and ACS [155].

Regarding long-term antiplatelet therapy, the PEGASUS-TIMI 54 trial examined two doses of ticagrelor (60 and 90 mg b.i.d.) vs. placebo in patients with a history of MI 1–3 years previously. The two ticagrelor doses each reduced, as compared with placebo, the rate of the efficacy endpoint (cardiovascular death, myocardial infarction, or stroke) with Kaplan–Meier rates at 3 years of 7.85% in the group that received 90 mg of ticagrelor b.i.d, 7.77% in the group that received 60 mg of ticagrelor b.i.d., and 9.04% in the placebo group [hazard ratio for 90 mg of ticagrelor vs. placebo, 0.85; 95% confidence interval (CI) 0.75–0.96; *P* = 0.008; hazard ratio for 60 mg of ticagrelor vs. placebo, 0.84; 95% CI 0.74–0.95; *P* = 0.004]. The 60 mg (but not the 90 mg) ticagrelor (plus aspirin) regimen also significantly reduced the stroke risk compared with aspirin monotherapy. The ticagrelor regimen was associated with a significantly increased bleeding risk. Patients with previous STEMI comprised more than 50% of the overall PEGASUS-TIMI 54 population, and subgroup analysis has shown consistent results in patients with previous STEMI vs. NSTEMI. Extension of DAPT beyond 1 year (up to 3 years) in the form of aspirin plus ticagrelor 60 mg b.i.d. may be considered in patients who have tolerated DAPT without a bleeding complication [156].

The studies mentioned above support the concept that duration of DAPT should be individualized as discussed in detail in the ESC/EACTS DAPT Consensus document [151].

#### Evidence from Japan

In the STOPDAPT prospective multi-center, single-arm study (*n* = 1525), 3-month DAPT after CoCr-EES implantation was compared with the prolonged DAPT regimen adopted in the historical control group from the RESET trial, where nearly 90% of patients had continued DAPT at 1 year [157]. A composite of cardiovascular death, MI, stroke, definite stent thrombosis and TIMI major/minor bleeding at 1 year occurred in 2.8 versus 4.0%, respectively (*P* = 0.06), and 3-month DAPT was considered at least as safe as the prolonged DAPT regimen. Interaction of acute myocardial infarction was not significant in the subgroup analysis (*P*_interaction_ = 0.65).

In the NIPPON trial (*n* = 3773), non-inferiority of net adverse clinical and cerebrovascular events (NACCE) (all-cause mortality, myocardial infarction, stroke, and major bleeding) of 6-month DAPT was shown as compared to 18-month DAPT following implantation of a Nobori DES with a biodegradable abluminal coating [158]. DAPT may be shortened according to patient’s ischemic and bleeding risks; however, these results should be interpreted with caution since the population in these trials was not limited to patients with acute myocardial infarction.

#### Recommendations

- DAPT in the form of aspirin plus prasugrel, clopidogrel or ticagrelor (e.g. clopidogrel should be used, if prasugrel or ticagrelor are not available or are contraindicated), is recommended for 12 months after PCI, unless there are contraindications such as excessive risk of bleeding.
- A PPI in combination with DAPT is recommended in patients at high risk of gastrointestinal bleeding.
- In patients with an indication for oral anticoagulation, oral anticoagulants are indicated in addition to antiplatelet therapy.
- In patients who are at high risk of severe bleeding complications, discontinuation of P2Y12 inhibitor therapy after 6 months should be considered.
- In STEMI patients with stent implantation and an indication for oral anticoagulation, triple therapy should be considered for 1–6 months (according to a balance between the estimated risk of recurrent coronary events and bleeding).
- In patients with LV thrombus, anticoagulation should be administered for up to 6 months guided by repeated imaging.
- The use of ticagrelor or prasugrel is not recommended as part of triple antithrombotic therapy with aspirin and oral anticoagulation.

## Treatment of non-infarct-related artery

### General recommendation in revascularization of non-infarct-related artery in acute MI

Management of non-infarct-related coronary arteries after primary PCI for ST-segment elevation Ml remains controversial. In the new guidelines released by the European Society of Cardiology in 2017 on the management of patients with ST-segment elevation Ml, complete revascularization for ST-segment elevation Ml patients with multivessel disease (MVD) was upgraded from III to IIa with level of evidence A.

In the Compare-Acute trial, 885 patients with ST-segment elevation Ml and MVD who underwent primary PCI were randomized in a 1:2 fashion to complete revascularization of non-infarct-related coronary arteries guided by FFR or no revascularization of non-infarct-related coronary arteries [159]. There was a significant reduction in MACCE at 1 year with FFR-guided complete revascularization (8 vs. 21%; *P* < 0.001). The benefit was mostly driven by a reduced risk of revascularization. Meta-analyses published so far on the topic do not incorporate the results of this study. In one of them focusing on the issue of timing for PCI of non-culprit artery lesions, which encompassed a total of 10 trials with 2285 patients, the reduction in the risk of cardiovascular events was observed irrespective of the timing of non-infarct-related coronary artery revascularization [160]. These results are thus in line with the 2017 ESC guidelines on ST-elevation Ml recommending ischemia-guided full revascularization [20].

In the setting of cardiogenic shock, the efficacy and safety of treating non-infarct-related coronary arteries in the context of primary PCI has been a matter of debate. In the Culprit Lesion Only PCI versus Multivessel PCI in Cardiogenic Shock (CULPRIT-SHOCK) trial (*N* = 706), the 30-day risk of a composite of death or severe renal failure leading to renal-replacement therapy was lower in patients who underwent initial PCI of the culprit lesion only compared with those who underwent immediate multivessel PCI [161]. In 2017 ESC guidelines, published 2 months before the publication of CULPRIT-SHOCK trial, Grade IIa recommendation with level of evidence C was applied for complete revascularization in ST-segment elevation Ml at patients with MVD who present with cardiogenic shock.

### Recommendations

- Routine revascularization of non-infarct-related artery (non-IRA) lesions should be considered in STEMI patients with multivessel disease before hospital discharge (either immediate or staged).
- Non-IRA PCI during the index procedure may be considered in patients with cardiogenic shock.

### Physiological assessment of non-infarct-related artery

FFR has been documented as a valuable tool to guide coronary intervention. The adenosine-free index, iFR, has emerged as a potential alternative to FFR. The Functional Lesion Assessment of Intermediate Stenosis to Guide Revascularisation (DEFINE-FLAIR) [162] (*N* = 2492) and Instantaneous Wave-free Ratio versus Fractional Flow Reserve in Patients with Stable Angina Pectoris or Acute Coronary Syndrome (iFR-Swedeheart) [163] (*N* = 2038) clinical trials both examined if iFR was non-inferior to FFR for PCI guidance. The primary end point in both studies was a composite of death from any cause, nonfatal Ml or unplanned revascularization at 1-year follow-up. In the DEFINE-FLAIR study, the primary end point occurred in 6.8% in the iFR group and in 7.0% in the FFR group (*P* < 0.001 for non-inferiority) [162]. In the iFR-Swedeheart study, the primary end point occurred in 6.7% in the iFR group as compared to 6.1% in the FFR group (*P* = 0.007 for non-inferiority). Moreover, iFR was associated with shorter procedural time and less procedural discomfort [163].

Recently, Quantitative flow ratio (QFR) was developed as an image-based index for estimating fractional flow reserve (FFR). In a retrospective, observational study conducted in Japan (*n* = 142) [164], QFR had good correlation (*r* = 0.80, *P* < 0.0001) and agreement (mean difference: 0.01 ± 0.05) with FFR. After applying the FFR cut-off ≤ 0.8, the overall accuracy rate of QFR ≤ 0.8 was 88.0%. On receiver-operating characteristics analysis, the area under the curve was 0.93 for QFR. In contrast, 3-D QCA-derived anatomical indices had insufficient correlation with FFR and diagnostic performance compared with QFR. An observational study to investigate diagnostic performance of QFR in comparison to FFR in intermediate stenosis in STEMI patients is on-going (NCT02998853).

In addition to FFR, iFR, QFR and CT-FFR could be useful tools to decide the treatment indication of non-infarct-related artery.

### Recommendations

- Physiological assessments should be considered before performing staged PCI in non-infarct-related artery.
